# Supplementary material for: Mass drug administration coverage and its determinants for the elimination of onchocerciasis in Ulanga District, Tanzania
Source: PLoS Negl Trop Dis. 2025 Apr 15;19(4):e0012219. doi: 10.1371/journal.pntd.0012219 (PMC12047752; doi:10.1371/journal.pntd.0012219)
Supplement: Attachment — Submitted filename: Response to Reviewers.docx [file pntd.0012219.s004.docx]

**REBUTTAL LETTER**

**For the manuscript “Mass drug administration coverage and determinants of drug uptake for elimination of Onchocerciasis” [PNTD-D-24-00690]**

**September 1, 2024**

**Reviewers' comments:**

**Methods**
*Reviewer #1:*

- *Are the objectives of the study clearly articulated with a clear testable hypothesis stated? YES*
- *Is the study design appropriate to address the stated objectives? YES*
- *Is the population clearly described and appropriate for the hypothesis being tested? YES*
- *Is the sample size sufficient to ensure adequate power to address the hypothesis being tested? YES*
- *Was correct statistical analysis used to support conclusions? YES*
- *Are there concerns about ethical or regulatory requirements being met? NO*

**Response:** We sincerely appreciate your careful review of our manuscript and your valuable feedback.

***Reviewer #2:***

*1) Village Screening and Confidence Intervals: Were all the villages screened during this survey? If not, it may be beneficial to provide 95% confidence intervals to understand the uncertainty around the mean coverage value found in each village, especially as the values are very close to the 85% coverage used as a threshold.*

**Response:** We sincerely appreciate your insightful comments regarding village screening and the presentation of confidence intervals in our survey and your attention to detail.

We would like to clarify that the screening was not carried out throughout the village. Systematic random sampling was employed to select household and within the household random sampling of the participants was conducted. We have re-analyzed the data to provide confidence interval that will help to understand the uncertainty around the mean coverage value found in each village. Also given the reference made from the African Program from Onchocerciasis control (APOC), we revised the threshold from 85% that was used in the initial manuscript to 80% recommended by WHO in the former APOC countries. We apologize for not including confidence interval in the manuscript and for using the threshold from Americas, causing any confusion.

*2) Study Area Details: In the first paragraph of the "Study Area" section, it would be beneficial to mention the year when Ov16 prevalence was determined in the district. Also, specify that the 20.7% prevalence mentioned on children is of Ov16.*

**Response:** The manuscript has been updated at first paragraph of the "Study Area" section to specify the year when the Ov16 prevalence was determined in the Ulanga District. We have also clarified that the 20.7% prevalence mentioned refers specifically to the prevalence of Ov16 in children. We appreciate your suggestions, as they will improve the clarity of the manuscript and provide readers with a more complete understanding of the study context.

*3) Specific Villages and Rural/Suburban Classification: The study area includes specific villages in Mahenge, specifically Isongo, Uponera, Mawasiliano, and Togo, as indicated in Figure 1. This should be clearly mentioned in the Methods section, not just in the abstract and results. Additionally, it would be beneficial to specify whether these villages are rural or suburban, as rural areas are typically more endemic for onchocerciasis. The methodology mentions that the district Ov16 prevalence stands at 76% in rural areas and 50% in suburban areas, which is relevant information.*

**Response:** Thank you for your thoughtful feedback regarding the inclusion of specific village details and their classifications in the "Methods" section of our manuscript. The "Methods" section has been updated to explicitly mention the specific villages included in our study: Isongo, Uponera, Mawasiliano, and Togo, as indicated in Figure 1. We have clarified the settlement characteristics of all the villages. It is now mentioned that all the four selected villages were in rural settings. We appreciate your suggestions for enhancing clarity and contextual understanding. We appreciate your recommendations, which we believe has contributed to the improvement we have made that improved the overall quality of our manuscript.

*4) Study Population Clarification: In the "Study Population" section, it would be useful to clarify what is meant by "participated in the MDA program for at least one (1) year." Does this mean they must have taken ivermectin for at least a year, been eligible for it for at least a year or been approached by a community drug distributer (CDD) for at least a year? Additionally, clarify that the study focuses on the coverage among eligible individuals (excluding pregnant women and children under 5), not the overall population. This distinction is important because WHO recommends 80% coverage of the overall population or coverage of all the eligible population, especially in historically hyperendemic settings such as Mahenge.*

**Response:** The study population section has been updated. The study population section has been clarified to include " must have been approached by a community drug distributor (CDD) during the last year before the study was conducted. On top of that we have clarified the stament to mention the study targeted the eligible population for Ivermectin MDA.

*5) Sample Size Estimate: In the "Sample Size Estimate" section, clarify whether the assumption that the proportion of drug uptake during MDA in Mahenge would be 78% refers to the overall population or the eligible population. This needs to be consistent with the study aims.*

**Response**: The sample size section has been updated to clarify that 78% refer to coverage of the of the total population.

*6) Data Management and Analysis Details: The methodology is mostly comprehensive. The "Data Management and Analysis" section could benefit from further detail. Specify which 95% level was used (e.g., confidence interval method, uncertainty interval, Wilson, Binomial, etc.). Additionally, clarify that "prevalence ratio**" refers to the prevalence of taking ivermectin during MDA and that it is an interchangable term with "relative risk". Specify which covariates were included in the Poisson regression (e.g., possible confounders such as age, gender, etc.). While this is partially mentioned in the results, it should be detailed in the methodology.*

**Response:** Thank you for your comments and we appreciate feedback on the robustness of the methodology for the study. You raise a valid point regarding adding further description of the Confidence interval used and the clarification of the prevalence ratio. We had included these details in the full report, we apologize for an overlook that led to an omission of the clarification when submitting the manuscript. We have updated the section to add more description as suggested.

*7) Statistical Methods: The choice of robust statistical methods is commendable. The authors' choice of regression methods, including the robust standard error, could be supported by reasoning either in the methodology or in the discussion as a potential strength and/or limitation.*

**Response:** Thank you, further descriptions on the factors that led to selection of poison regression over other regression analysis has been added in the methods section.

*Reviewer #3: partly, see my general comments*

**Response:** We are thankful for your exhaustive review of our manuscript and your valuable feedback. We have addressed all observations you have highlighted your general comments.

**Results**

***Reviewer #1:***

- *Does the analysis presented match the analysis plan? YES*
- *Are the results clearly and completely presented? see my general comments*
- *Are the figures (Tables, Images) of sufficient quality for clarity? Some changes needed*

**Response:** We are thankful for your in-depth review of our manuscript and your valuable feedback. We have addressed all the changes suggested in your general comments.

***Reviewer #2:***

*1. Clarification of "±15 years": In "participants was 37.8 ±15 years," please define what "±15 years" represents.*

**Response:** Thank you for raising a valid point, we had included these details in the full report, unfortunately we overlooked when developing this manuscript. We have updated the section to add more description that clarifies that "±15 years" is a standard deviation.

*2. Correction in Referencing: Instead of referencing "Figure 1," it should be "Table 1."*

**Response:** References in figure 1 have been updated as suggested.

*3. Clarification of WHO Coverage Recommendations: The statement "Drug uptake coverage from all studied villages was below the optimal coverage (85%) recommended by WHO for successful transmission interruption and elimination of Onchocerciasis" needs clarification. Since the coverage in question pertains to the eligible population for taking ivermectin, I ponder if the recommended coverage should be nearly 100% of the eligible population or 80% of the overall population (including those not eligible for ivermectin, such as pregnant women, lactating women during the first week after birth, children under 5 years of age, and seriously ill patients)? If the authors want to keep the 85% threshold, then appropriate references should be mentioned.*

**Response:** Thank you for your careful review and valuable feedback. We would like to clarify that given the reference made from the African Program from Onchocerciasis control (APOC), we revised the threshold from 85% that was used in the initial manuscript to 80% recommended by WHO in the former APOC countries, we have also included corresponding reference. We have also updated the section to clarify that the coverage mentioned in the manuscript refer to the 80% of the overall population. We apologize for an oversight in the manuscript that led to the confusion observed.

*4. Define Abbreviations in Tables: Define any abbreviations used in the tables, such as PR (Prevalence Ratio) and MDA (Mass Drug Administration) in Table 1. Also, clarify what "*" signifies, typically indicating statistical significance. Similarly, define CPR, CI (Confidence Interval), and APR (Adjusted Prevalence Ratio) in Table 2.*

**Response:** All tables have been updated and all abbreviations defined as suggested.

*5. Clarification on Drug Uptake: The statement "In Isongo village, drug uptake was 2.4 times higher in 2017 [PR = 2.41 (2.2-2.3)], while drug uptake in 2018 was 1.2 times higher [PR = 1.18 (1.1-1.3)] compared to that of 2016 respectively" seems inconsistent with the proportions shown in Table 1, which are lower for 2017 and 2018 than for 2016. This discrepancy may relate to the relative risks produced from Poisson Regression (PR), but even if that is true, would it be correct to state that the drug uptake was higher? Please clarify this.*

**Response:** This was wrongly interpreted here and has been rephrased to reflect the correct result interpretation.

*6. Correcting Abbreviation Usage: In "OR = 0.87(0.78-0.96)," should "OR" be "PR"? Please define the abbreviation the first time it is used in the text. Also, define APR.*

**Response:** The abbreviation OR has been corrected to read PR and both PR and APR abbreviations have been defined as suggested.

*7. Improving Comparisons: Improve and complete the comparison in the sentence: "Participants aged 15-24 and 25-34 years were 3 times [APR = 2.8 (95% CI: 1.3-6.2)] and 2 times [APR = 2.3 (95% CI: 1.04-4.9)] more likely to take ivermectin, respectively, compared to the adult population aged 45-54 (Table 2)."*

**Response:** The manuscript has been updated. The comparison has been completed to "Younger age groups (15-24 and 25-34) had significantly lower drug uptake compared to the 45-54 age group, even after adjusting for other factors. Older adults (55+) did not show significant differences in drug uptake”.

*8. Consider Merging Categories: In the regression analysis shown in Table 2, consider merging categories for rare values such as "college," "none," and "student." Small sample sizes in these categories might compromise the model output. A rule of thumb is to have at least 10 observations per category per level (yes/no).*

**Response:** Thank you for raising an important observation. We have merged the categories with less than 10 observations and re-analyzed to update the model output and understand the contribution of education into community drug uptake.

*9. Clarification of Variables in Table 3: Clarify the variable "Perceived reason for drug uptake" in Table 3 (and correct the table's label from Table 2 to Table 3). What is meant by "prevention effects" and "side effects"? It appears that those who perceived ivermectin as preventive took it less often than those who perceived it as having possible side effects. Also, it is noteworthy how most of those who feared taking ivermectin with alcohol still decided to take the drug. Please provide more context and clarification.*

**Response:** Thank you for your deep review and valuable observation of the study results. We have revised the interpretation in commensurate with comments raised to make the interpretation sound. Further clarifications of the variable *“Perceived reason for drug uptake*” and *"side effects"* have been provided to mean the following: *“Perceived reason for drug uptake*” = the reason that led the participant to whether swallow Ivermectin (IVM) or refrain from taking and swallowing IVM during CDTI), and *"side effects" =* impact of previous IVM side effect in taking IVM and swallowing during CDTI. We apologize for an oversight in the manuscript, causing any confusion caused.

***Reviewer #3:***

*Partly, see my general comments*

**Response:** We are grateful for your review valuable feedback. We have addressed all the changes suggested in your general comments

**Conclusions**

***Reviewer #1:***

- Are the conclusions supported by the data presented? YES
- Are the limitations of analysis clearly described? Not sufficient
- Do the authors discuss how these data can be helpful to advance our understanding of the topic under study? Old data is limited their relevance
- Is public health relevance addressed? Limited relevance today because old data

**Response:** We are grateful for your review of our manuscript and your valuable feedback. We have made significant review of the conclusion section linking it to the current literature and programmatic update in Tanzania on Onchocerciasis elimination intervention in Tanzania. Given the current OV status in the area and the update we have made we believe this study will be relevant in adding values to the current body of knowledge on Onchocerciasis elimination and CDTI implementation.

***Reviewer #2:***

*1. Commendation and Clarification on MDA Coverage: I commend the authors on the discussion around MDA coverage and persistent onchocerciasis prevalence and presumable transmission. However, the MDA values seem to be reaching a likely maximum feasible value in the villages. Considering the population usually excluded from receiving ivermectin (pregnant women, lactating women during the first week after birth, children under five, and very sick individuals), the coverage cannot go much higher. Being that Ulanga is one of the most historically endemic settings for onchocerciasis in Tanzania, biannual CDTI is possibly needed to halt transmission. This is indeed a challenge that many settings might face as they approach the elimination goal where the blackfly vector is highly prevalent.*

**Response:** We appreciate the good feedback. Given the findings from various studies and programmatic evaluation. The biannual CDTI was initiated in Mahenge focus.

*2. Emphasis on Additional Control Interventions: Instead of solely emphasizing the need to intensify MDA awareness campaigns targeting less compliant groups in the community to reinforce the benefits of ivermectin in onchocerciasis control and address the community misconceptions about MDA, which might only increase coverage by 5-10% if extremely successful, it might be more effective to advocate for additional control interventions, such as the current biannual CDTI, taking into account the historically high onchocerciasis endemicity of Mahenge and the long history of ivermectin annual delivery.*

**Response:** We are grateful for your review for the insightful feedback. We have reviewed the conclusion section to include emphasis for other public health interventions as suggested including improving delivery of the annual CDTI, community mobilization towards slash and clear to reduce flies breeding sites and investing on community understanding on control and prevention of the diseases.

*3. Recent Changes and Comparisons: It was not mentioned that Mahenge recently switched from annual to biannual MDA to further control onchocerciasis. This should be stated. Additionally, referencing recent papers on CDTI coverage in Mahenge for comparison would be very beneficial and expected. An example could be the paper: "Impact of a bi-annual community-directed treatment with ivermectin programme on the incidence of epilepsy in an onchocerciasis-endemic area of Mahenge, Tanzania: A population-based prospective study."*

**Response:** The manuscript has been updated. It is now mentioned introduction of biannual CDTI as suggested which was initiated a year before this study was conducted. We have also cited several new that literature on the subject matter in the areas including the one you have suggested to add power to the manuscript submitted.

*4. Association Between Onchocerciasis and Epilepsy: Given the association between onchocerciasis and epilepsy, known as onchocerciasis-associated epilepsy (OAE), where the onset of epilepsy occurs in children and teenagers aged 3 to 18 years, ivermectin intake has been associated with a decrease in epilepsy incidence in these age groups. This highlights the importance of achieving the highest coverage of ivermectin in the group at risk of developing onchocerciasis-associated epilepsy in Mahenge. This is particularly relevant as many onchocerciasis-endemic sites with high epilepsy, including nodding syndrome, prevalence have the lowest ivermectin coverage in younger age groups. Mahenge was indeed the first place to report the public health problem of nodding syndrome in 1962.*

**Response:** We appreciate the feedback, we acknowledge that during literature review we reviews number of studies on the association between and OV and nodding syndrome, and we included synthesis of those literature in the research report. We have revised the manuscript to include a snapshot of these relation into this study.

*5. Study Limitations: The study limitations should be discussed before the conclusion.*

**Response**: The manuscript has been revised and limitation section has been moved above the conclusion.

*6. Discussion of Other Potential Avenues to Increase Coverage: Being a paper exploring the limitations of ivermectin coverage in the population, it would be valuable to discuss other potential avenues to increase coverage:*

**Response**: Thank you, the manuscript has been revised discussion on the alternative solutions to increase coverage has been included in the revised document.

*6.1) Moxidectin:* *Another microfilaricidal drug like ivermectin, moxidectin, can be given to children aged 4 years old, complementing ivermectin, which can only be given to children aged five and above. This could help increase coverage among younger children.*

**Response**: Thank you for the insightful review and feedback. You raise an important point regarding adding discussion on other potential avenue for increasing coverage especially application of moxidectin in children. It is unfortunate that despite having a detailed section of this information in the literature review of the main study report, it was overlooked when drafting this manuscript. We have made a review of the conclusion section to include this suggestion you have made.

6*.2) Interventions for Pregnant Women: Exploring potential interventions for* *pregnant women, who can pass years excluded from ivermectin delivery and represent infection reservoirs, could also be discussed.* *Potential strategies could include the development of alternative treatments safe for pregnant women or targeted interventions post-pregnancy to ensure they receive ivermectin as soon as it is safe to do so.*

**Response**: Thank you for the insightful suggestion, we have revised the manuscript and included the discussion on the strategies to reach more women who missed CDTI due to pregnancy. The strategies discussed include the application of Gender Equity and Social Inclusion (GESI) using the IDEA methodology that ensures prospective follow-up of cohort that missed last CDTI, educate and sensitize them to participate in the next CDTI.

***Reviewer #3:*** *yes*

**Response**: Thank you, and we are grateful for your consideration.

**Editorial and Data Presentation Modifications?**

***Reviewer #1:***

*Old data with limited relevance. Maybe acceptable if more recent information about the study area is added.*

**Response**: Thank you for feedback and consideration. We have made a significant review of the manuscript and cited the most recent information on the subject matter. We have run some new analysis based on the recommendation we received to align to both current need and reviewers’ suggestion.

***Reviewer #2:***

*Missing Reference Numbers: The study appears to have references with numbers up to 72, with many missing numbers in the middle. Additionally, the reference list only goes up to 24. Please ensure that all references are correctly numbered and included in the reference list.*

*I recommend a thorough review of the entire document for language and punctuation. While the content is generally clear, certain passages could benefit from improved clarity and precision. For instance, here are some abstract corrections:*

*1.1. Correct "Onchocerciasis remain to be an important" to "Onchocerciasis remains an important."
1.2. In the sentence "However, current reports indicate high prevalence of Onchocerciasis in both human and vector species probably because of poor treatment coverage indicating limited evidence for transmission," two points need clarification:*

*- It seems that poor treatment coverage is being identified as the cause of limited evidence for active transmission.
- The phrase "limited evidence for transmission" likely means the opposite of what is intended. It seems the authors want to express that* *there is* *preliminary evidence of persistent onchocerciasis transmission, which likely sustains the observed high prevalence.*

*1.3. Add a comma after "Morogoro" in "Ulanga District, Morogoro, Tanzania from April-June 2019." Remove the space in "25.5 %" and add a comma after "Mawasiliano (68%)."*

*1.4. In "Low coverage below the WHO optimal recommended coverage," the words "low" and "below" are redundant. I suggest removing "low."*

**Response**: Thank you for the detailed review, observation and feedback. We acknowledge the missing numbers in the reference. We would like to clarify that we had significant citation on the literature review section of this study, it is unfortunate that we overlooked some of the reference we made as we were when drafting this manuscript. We have made a review of the entire document, added more recent references with correct numbering. All reference has been added to the refence list. Similarly, all recommended edits for improving clarity and precision have been accepted and all phrases and sections have been edited as suggested.

***Reviewer #3:*** *major revision*

**Response**: Thank you for feedback, recommendation and consideration. We have made significant review of the manuscript as recommended.

**Summary and General Comments**

***Reviewer #1:***

*The papers address an important challenge for the elimination of onchocerciasis in Tanzania: to obtain a sufficient high uptake of ivermectin.*

*The paper is well written, and the results are interesting. However, the main problem with the paper is that the study was done in 2019. This limits the relevance of the data for operational use because we need to know what the ivermectin coverage is today, in 2024. In the meantime, in the study area CDTI has been switched from annual to bi-annual distributions. This certainly had an effect on the current uptake of ivermectin in the area. It also had an effect on the incidence of epilepsy in the area. Important to mention this in the discussion. See ref “Bhwana D, Amaral LJ, Mhina A, Hayuma PM, Francis F, Siewe Fodjo JN, Mmbando BP, Colebunders R. Impact of a bi-annual community-directed treatment with ivermectin programme on the incidence of epilepsy in an onchocerciasis-endemic area of Mahenge, Tanzania: A population-based prospective study. PLoS Negl Trop Dis. 2023 Jun 28;17(6): e0011178.*

*I propose to explain better why it was important in 2019 to investigate ivermectin coverage in the Ulanga District in Tanzania and not in other onchocerciasis endemic areas in Tanzania.*

**Response**: Thank you for a detailed review and feedback. We have updated the manuscript to add more description of the discussion section to include the effects of biannual CDTI on the coverage and disease elimination efforts. We have also added the relevance of studying IVM coverage in Ulanga districts. review of the manuscript as recommended.

*The authors report the 2009 and 2017 survey data in Ulanga. However, it is important to specify that some of the data were obtained in selected rural villages, different from the villages investigated for ivermectin coverage in 2019.*

**Response**: Manuscript has been updated and provided that specific description has been provided.

*On the map Figure 1 it would be interesting to indicate the localisation of the 2019 study villages together with those investigated in the epilepsy prevalence surveys in 2017-18.*

**Response**: We have updated the document and an explicit description of the village localization has been provided as suggested.

*A limitation of the study is that children < 15 years of age were not included. This is important because they are at risk for developing onchocerciasis-associated epilepsy and therefore it is important that a high ivermectin coverage is reached in the age group.*

**Response**: Thank you, the suggestions have been updated in the revised document.

**Methods**
*Little is said about the questionnaire and how it was used. How were the house visits done? What happened if in a selected household nobody was home? Secondary data collection was done in 2 health facilities. Maybe I missed it, but I do not see the results clearly explained in the text. Was the data that were obtained different from those obtained by interviewing the study participants?*

**Response:** Thank you for your comments and we appreciate your feedback on the methodology. You raise a valid point regarding application of the questionnaire and the how household participants were enrolled in the study. We had included these details in the full report, we apologize for an overlook that led to an omission of the clarification when submitting the manuscript. We have updated the section to add more description as suggested.

**Minor comments**

*Multi variate analysis should this be multivariable analysis?*

**Response:** Yes

*Table 1 Togo should be written in bold*

**Response:** Corrected as suggested

*All Tables: Harmonise spaces between numbers and percentages*

**Response:** Edited and harmonized s suggested

*Table 2. Onchocerciasis Targeted health education: not clear what the following variables stand for:*

*Hygiene and Ocho? Other but not Ocho?*

**Response:** The following are the meaning of the variables “*Hygiene and Ocho”* Implies health education cession in onchocerciasis MDA messages was part of the hygiene education programs in the area. *Other but not Ocho?* implies health education cession that did not mention onchocerciasis at all.

*I propose to add the questionnaire as supplementary material to better understand these variables.*

**Response:** The questionnaire has been submitted as a supplement document as requested.

***Reviewer #2:***

*The study by Mhiche et al. addresses a crucial public health issue by examining mass drug administration (MDA) coverage and the factors influencing drug uptake in onchocerciasis-endemic regions. The research demonstrates a thorough understanding of onchocerciasis and its elimination guidelines, providing valuable insights into the challenges and successes of MDA programs.*

*While the study is comprehensive and informative, several areas require improvement to enhance clarity and accuracy. These include detailed explanations of the methodology, addressing discrepancies in the results, incorporating recent changes in MDA strategies, and ensuring all references are correctly numbered and included.*

*With these revisions, the manuscript could make a significant contribution to onchocerciasis research. I acknowledge the authors' hard work and dedication to improving public health outcomes.*

**Response**: Thank you for feedback, recommendation and consideration. We have made significant review of the manuscript as recommended.

**1) Abstract remarks:**

*1.1) Clarification of "±15 years": Please define what "±15 years" represents in the result section of the abstract. Is this a standard deviation, or another statistical measure?*

**Response:** We have updated the section and clarifies that "±15 years" is a standard deviation.

*1.2) WHO Optimal Coverage: The statement "below the optimal coverage recommended by WHO (85%) for successful transmission interruption" needs clarification. From my understanding, the WHO guideline for elimination is to deliver ivermectin to at least 80% of the population. However, in the Americas, the guideline specifies at least 85% of the eligible population. If you are referring to the latter, please clarify that you are working with the eligible population and not the overall population. However, keep in mind that recommendations for the Americas might not be applicable to sub-Saharan Africa, as the latter have more competent vectors and, therefore, higher endemicities.*

**Response:** We revised the threshold from 85% that was used in the initial manuscript to 80% recommended by WHO in the former APOC countries.

1*.3) Terminology in Association: In the phrase "decreased drug uptake [APR = 12(95% CI: 2.4-60.9), p<0.05)] was attributable," the word "attributable" implies causation, which cannot be formally assessed with a Poisson regression. I suggest using terms like "associated with" or "linked to" instead.*

**Response:** We revised the interpretation and used the terminology suggested.

*1.4) Correct "Adjusted Prevalence Ration" to "Adjusted Prevalence Ratio". Also, consider that the terminology "adjusted relative risk" is more often used.*

**Response:** We have corrected the phrase and changed to use "adjusted relative risk" as suggested.

**2) Introduction Remarks:**

*2.1) Clarification of Geographic Focus: The phrase "and in Tanzania in particular" should be corrected to "**including Tanzania" to avoid implying that onchocerciasis in Africa is particularly prevalent in Tanzania.*

**Response:** We have corrected the phrase and changed to remove the statement including Tanzania as suggested.

*2.2) Detailed Explanation of Epilepsy Co-Endemicity: In the last paragraph of the introduction, epilepsy is briefly mentioned as co-endemic with parasitic infections such as onchocerciasis. A more detailed explanation and referencing would be beneficial. Specifically, Mahenge is one of the most well-known foci demonstrating the strong association between onchocerciasis and epilepsy, including nodding syndrome. In Tanzania, Mahenge is clearly the most well-known focus for nodding syndrome. This context can support the authors' argument that the high prevalence and incidence of epilepsy in the area may further indicate active transmission of onchocerciasis. It has been shown in several endemic settings, including Mahenge, that the implementation and strengthening of onchocerciasis control efforts are followed by a significant decrease in the incidence of epilepsy, including nodding syndrome. Providing this detailed explanation and referencing relevant studies will strengthen the introduction and support the study's rationale.*

**Response:** We acknowledge that during literature review we reviewed number of studies on the association between and OV and nodding syndrome, and we included synthesis of those literature in the research report which included statement like **“****One study revealed increased prevalence of Epilepsy (nodding Sundrome) that implies presence of relatively high level of filaria worms in the rea, suggesting limited progress made in the control of Onchocerciasis in Ulanga”.** We have revised the introduction section to include a snapshot of these relation into this study.

*2.3) Historic Prevalence Information: Including specific information, with numbers if available, on historic prevalence of Mahenge would provide valuable context for understanding the current study findings. It would help readers appreciate the progress made and the challenges that remain in controlling onchocerciasis.*

**Response**: We have made a significant review of the manuscript as recommended. Historic data has been added as suggested.

**Reviewer #3: Comments**

*1. Under abstract section how the determinants were explored, better to use other appropriate term*

**Response:** Thank you for your suggestion. However, the term determinants were used to mean factors that determine MDA uptake in the study area. We have added further description to increase clarity and understanding.

*2. Correct some typos throughout the paper*

**Response:** Corrected as suggested

*3. Why the study was this much late, are there no changes since that (2019).*

**Response**: Thank you, we understand that the study is late, however we made dissemination of the finding to several stakeholders and given the positive feedback we got, we found it high time we make the study available to the scientific community. Few changes have happened, and we have discussed the new literature in our discussion.

*4. Eligibility criteria is not clear, what was sampling and study unit, how many from each household?*

**Response:** Thank you for your comments and we appreciate your feedback on the methodology. You raise a valid point regarding clarity of the eligibility criteria, and sampling unit. We had included these details in the full report, we apologize for an overlook that led to an omission of the clarification when submitting the manuscript. We have updated the section to add more description to increase clarity.

*5. Regarding the sample, what does 78% as previously determined mean?*

**Response:** The statement “78% as previously determined” *MDA coverage as it was determined from the community survey.* We apologize if this was not explicitly clarified in the manuscript, causing any confusion.

*6. How were 2 data collection methods (primary and secondary) used together?*

**Response**: Thank you for your question regarding the integration of primary and secondary data collection methods. The combination of these two approaches was central to our study design, allowing us to triangulate data for a more robust analysis.

**Primary Data Collection:**

- The primary data consisted of data collected directly from participants through surveys and key informant interviews. This data was collected to obtain firsthand, up-to-date information on variables relevant to our research question, such as participant behaviours, experiences, and insights that were not available in secondary sources.

**Secondary Data Collection:**

- Secondary data was gathered from CDTI community register and Health Facility summary data form. These data sources were used to provide a broader context for the study, including background statistics, historical trends, and comparative benchmarks.

The combination allowed us to address gaps that might have been left if only one type of data was used. Primary data provided direct, current information from respondents, while secondary data ensured that our study was grounded in a broader context, offering greater depth and validation. By integrating both primary and secondary data, we were able to enhance the robustness of our findings and produce a more comprehensive understanding of the subject matter. These details were explicitly discussed in the main report, however, for the purpose of this submission we aimed to report results from the community survey that aimed to explore factors that determine community participation in CDTI. We have therefore revised the documents, all other information that was not intended to be submitted has been removed from this new document. We apologize for an overlook and not excluding this from initial submission and causing confusion. We hope this clarifies the rationale behind using both data sources

*7. Did you included qualitative data, “in addition, an interview guide was developed to guide the key informant interviews.”, if so how, for what purpose, who were the participants….?*

**Response**: Thank you for raising this point. We did indeed include qualitative data in our study to complement the quantitative findings and provide a more comprehensive understanding of the subject.

*8. Why was this analysis selected, ‘Following descriptive analysis, generalized linear model with modified Poisson regression was used to estimate prevalence ratio.’ And did the assumptions were checked and if so give us supplementary files*

**Response**: Thank you for raising a valid concern. We would like to clarify that after a thorough literature review based on the information we have collected. The generalized linear model (GLM) with modified Poisson regression was chosen for the following reasons.

We aimed to estimate prevalence ratios (PR), which are more appropriate than odds ratios (OR) for cross-sectional studies, particularly when the outcome is common. Modified Poisson regression with a log link function is a well-established method for this, as it directly provides PR estimates, avoiding the issue of overestimating risk with ORs. This method also handles binary outcomes effectively and provides robust estimates, especially in cases of non-rare outcomes.

Likewise, we ensured that all assumptions of the GLM were checked before applying the model. Specifically, we assessed for: Linearity of independent variables in the log link of the model, No perfect multicollinearity among the independent variables and the distribution of residuals was also examined to ensure model fit.

We have included the results of these checks in the supplementary files as requested. We hope this addresses your concern, and we would be happy to provide further clarifications if needed.

*9. What is your base for your categorization for variables like age, occupation, etc.*

**Response**: Thank you for your question regarding the categorization of variables such as age and occupation. The basis for categorizing these variables was carefully considered to align with standard practices, facilitate meaningful analysis, and ensure comparability with existing studies. The categorization was mainly influenced by the distribution of data. Categories were created to ensure enough participants in each group for meaningful statistical analysis, avoiding too many small or empty categories. On top of that, we used cut-offs that reflect social and health-related differences observed in previous research, ensuring our findings could be interpreted in a relevant and actionable manner. We hope this explanation clarifies our approach to categorizing the variables.

*10. Title needs revision to make concise, what if Mass drug administration coverage and its determinants…. think critically over it*

**Response**: Thank you for your suggestion regarding revision of the title. We have revised the tittle to reas as “**Mass drug administration coverage and its determinants for elimination of Onchocerciasis in Ulanga District”** This title emphasizes the core focus of the study which is the determinants of both coverage and uptake of mass drug administration (MDA) while also retaining the geographical context of Ulanga District and the disease focus on Onchocerciasis elimination.

*11. Remove repetition from the abstract, remove interpretation from result*

**Response:** Corrected as suggested

*12. How you see this finding with very wide confidence interval like (CI:2.9-60.9) ….*

**Response:** Thank you for your important question regarding the interpretation of the wide confidence interval (CI: 2.9-60.9) associated with our findings as papered in table number 2 of the findings. While the wide confidence interval highlights the need for cautious interpretation, it also underscores the importance of further research to clarify the relationship and refine our understanding. We have added these observations in the reviewed document.

*13. Why was Modified Poisson regression chosen compared to others?*

**Response:** Thank you for your question regarding our choice of Modified Poisson regression for our analysis. This method was selected for various important reasons:

Nature of the Outcome Variable:

- Our study involved a binary outcome (e.g., drug uptake: yes/no), where prevalence ratios (PR) rather than odds ratios (OR) were more appropriate. Modified Poisson regression is particularly suitable for estimating PRs in the context of common outcomes, as it provides a direct estimation of prevalence without the limitations of ORs, which can overestimate risk when the outcome is prevalent.

Handling of Common Outcomes:

- In studies with common outcomes, the use of traditional logistic regression can lead to biased OR estimates that do not accurately reflect the underlying prevalence. The Modified Poisson approach mitigates this bias by modelling the data more directly, allowing smooth results interpretation in public health contexts where understanding prevalence is critical.

Robustness to Assumptions:

- Modified Poisson regression is robust and accounts for overdispersion in the data, which can occur if the variance of the outcome exceeds the mean. This is particularly relevant in our dataset, where variability in drug uptake could influence the results.

Simplicity and Interpretability:

- The interpretation of results from Modified Poisson regression is straightforward. The output provides prevalence ratios that are easy to communicate to stakeholders and practitioners, making it a practical choice for studies aiming to inform public health interventions.

Alignment with Study Goals:

- Our research aimed to identify and understand the determinants influencing drug uptake for Onchocerciasis elimination. Modified Poisson regression aligns well with this goal, as it allows for a clear assessment of how various factors impact the likelihood of drug uptake in the population.

Therefore, we believe this method provided the most appropriate framework for our analysis.

*14. Better to put sampling procedure in figure to make it clear for readers by showing the stages*

**Response:** Thank you for your valuable suggestion regarding the presentation of the sampling procedure. We agree that visual representations can enhance clarity and improve reader understanding of complex processes. We have revised the manuscript to include this figure, ensuring that it is well-integrated with the text and appropriately referenced. We believe this addition has significantly enhanced the methodological clarity of our study.

*15. Did qualitative method included? How? The purpose, if so, how the data was analyzed and interpreted?*

**Response:** Thank you for your inquiry regarding the inclusion of qualitative methods in our study. Yes, qualitative methods were indeed incorporated to enrich our understanding of the factors influencing drug uptake for Onchocerciasis elimination. however, for the purpose of this submission we aimed to report results from the community survey that aimed to explore factors that determine community participation in CDTI. We have therefore revised the manuscript, all other information that was not intended to be submitted has been removed from this new document. We apologize for an overlook and not excluding this from initial submission and causing confusion. We hope this clarifies the rationale behind using both data sources

**There is a statement w/c talk about KII.**

*16. Measurement and operational definitions should be presented clearly?*

**Response:** Thank you for your valuable feedback regarding the clarity of measurement and operational definitions in our study. We appreciate your suggestion and are committed to improving the presentation of our study's measurements and definitions. We have revised the manuscript to include all key definitions in a dedicated section that clearly outlines each operational definition we have used in our study.

*17. Discussion lacks depth, there are also incomplete sentences, …need major revision*

**Response:** Thank you for your constructive feedback regarding the depth of the discussion section and the presence of incomplete sentences. We recognize the importance of a thorough and well-articulated discussion to effectively communicate the significance of our findings. We have undertaken a major revision of the discussion section to provide a more comprehensive analysis of our results.

*18. Clearly justify way to control bias, better to include strobe checklist*

**Response:** Thank you for your insightful feedback regarding the need to clearly justify the measures taken to control bias in our study and the suggestion to include the STROBE (Strengthening the Reporting of Observational Studies in Epidemiology) checklist.

We recognize that controlling bias is essential to ensure the validity of our findings. To address potential biases, we implemented several strategies, including:

- **Random Sampling:** We utilized a four-stage cluster and a systematic random sampling to draw the household to be involved on the study. While at the household, the simple random sampling was used to select participants for our study. This minimized selection bias and ensured that the sample is representative of the broader population in Ulanga District.
- **Standardized Instruments:** We employed standardized survey instruments and interview guides, which provided consistent measures across all participants, further reducing variability due to measurement bias.
- **Data Verification:** We conducted follow-up checks on the collected data to identify and correct any inconsistencies or errors, enhancing data reliability.

We had included the detailed description in the main report. For the purpose of this submission, we overlooked some of the details. Therefore, we have revised the methodology section to include a detailed description of the bias control measures implemented in our study. Additionally, we ensured that the STROBE checklist is complete and accurately reflects our study's design and conduct.

--------------------

PLOS authors have the option to publish the peer review history of their article ([what does this mean?](https://journals.plos.org/plosntds/s/editorial-and-peer-review-process#loc-peer-review-history)). If published, this will include your full peer review and any attached files.

If you choose “no”, your identity will remain anonymous but your review may still be made public.

Do you want your identity to be public for this peer review? For information about this choice, including consent withdrawal, please see our [Privacy Policy](https://www.plos.org/privacy-policy).

Reviewer #1: Yes: Colebunders Robert

Reviewer #2: Yes: Luís-Jorge Amaral

Reviewer #3: No

**Figure Files:**

While revising your submission, please upload your figure files to the Preflight Analysis and Conversion Engine (PACE) digital diagnostic tool, https://pacev2.apexcovantage.com. PACE helps ensure that figures meet PLOS requirements. To use PACE, you must first register as a user. Then, login and navigate to the UPLOAD tab, where you will find detailed instructions on how to use the tool. If you encounter any issues or have any questions when using PACE, please email us at [figures@plos.org](mailto:figures@plos.org).

**Data Requirements:**

Please note that, as a condition of publication, PLOS' data policy requires that you make available all data used to draw the conclusions outlined in your manuscript. Data must be deposited in an appropriate repository, included within the body of the manuscript, or uploaded as supporting information. This includes all numerical values that were used to generate graphs, histograms etc.. For an example see here: <http://www.plosbiology.org/article/info%3Adoi%2F10.1371%2Fjournal.pbio.1001908#s5>.
 
**Reproducibility:**
To enhance the reproducibility of your results, we recommend that you deposit your laboratory protocols in protocols.io, where a protocol can be assigned its own identifier (DOI) such that it can be cited independently in the future. Additionally, PLOS ONE offers an option to publish peer-reviewed clinical study protocols. Read more information on sharing protocols at <https://plos.org/protocols?utm_medium=editorial-email&utm_source=authorletters&utm_campaign=protocols>

__________________________________________________
In compliance with data protection regulations, you may request that we remove your personal registration details at any time. (Use the following URL: <https://www.editorialmanager.com/pntd/login.asp?a=r>). Please contact the publication office if you have any questions.
