# Supplementary material for: Mass drug administration coverage and its determinants for the elimination of onchocerciasis in Ulanga District, Tanzania
Source: PLoS Negl Trop Dis. 2025 Apr 15;19(4):e0012219. doi: 10.1371/journal.pntd.0012219 (PMC12047752; doi:10.1371/journal.pntd.0012219)
Supplement: Attachment — Submitted filename: Response_to_Reviewers_auresp_2.docx [file pntd.0012219.s005.docx]

**REBUTTAL LETTER**

**For the manuscript “Mass Drug Administration Coverage and Its Determinants for the Elimination of Onchocerciasis in Ulanga District” [PNTD-D-24-00690]**

**March 2025**

**Reviewers' comments:**

**Methods**

***Reviewer #2:***

*Please mention the reference where this important information was obtained: "The district OV16 prevalence as of 2017 stands at 76% and 50% in rural and suburban areas respectively."*

**Response:** Thank you for your comment. The OV16 prevalence data for 2017 (76% in rural areas and 50% in suburban areas) is sourced from the 22nd reference in the manuscript, a study conducted by Mbando et al. We have added the citation for proper attribution.

***Reviewer #2:***

*Remove either in " These villages were classified as either rural, where onchocerciasis prevalence tends to be higher (Figure 1)".*

**Response:** Thank you for your suggestion. "We have removed 'either' for clarity."

**Results**

***Reviewer #2:***

1. *Table 1, How are the numbers of people surveyed in each village higher than the study sampled population of 502 participants? Please clarify this in the methodology/results where this data comes from reference it if applicable*

**Response:** Thank you for your comment. The explanation about the secondary data collected in each village is clearly outlined in the data collection section of the methodology. However, to improve clarity, additional information has been added to further clarify the data source and ensure consistency.

2. *Should be "crude" in "CPR= Cruse Prevalence Ratio"*

**Response:** Thank you for pointing that out. It should indeed be "Crude" instead of "PR only." We have made the correction accordingly.

**Conclusions**

***Reviewer #2:***

*1. It should be mentioned in the study that Mahenge is currently under biannual MDA (ref 16), which this study results support.*

**Response:** Thank you for the suggestion. W agree that it would be important to mention that Mahenge is currently under biannual MDA (ref 16), and how the study's results support this. We have made correction accordingly.

*2. I am confused between staying that two of the 4 villages met the 80% WHO recommended coverage for onchocerciasis elimination, but then, in table 1, all of them have <<80% coverage on the population data. As stated above, I am also still confused between the data collected from the 502 participants (2019) and the one collected from a larger population (Table 1, 2016-2018). This should be clarified. Does the study seem to suggest that coverage increased between 2016-2018 and 2019? The coverages of table 1 (2016-2018) and the participants in 2019, being from the same villages and assuming independence, should be statistically analysed in the results and interpreted in the discussions, otherwise, table 1 seems to be of little to no relevance for the discussion of this manuscript.*

**Response:** Thank you for your thoughtful comment and we greatly appreciate your feedback. we understand the confusion between the 80% WHO recommended coverage, and the data presented in Table 1. Here's a clarification to improve the manuscript’s clarity and robustness:

1. **Coverage Discrepancy**: The statement that two of the four villages met the 80% coverage target refers to the specific time period or data point where this was observed. However, Table 1 presents population-level data over a broader timeframe (2016-2018), which likely reflects variations in coverage during that period, thus showing all villages with coverage below 80%. These data were from the secondary data review which were collected from Community drug distribution (CDD) registers for the three years period. Details explanation has been provided in the methodology section.
2. **502 Participants (2019) vs. Larger Population (2016-2018)**: You are correct in noting the potential inconsistency between the data sets from 502 participants in 2019 and the larger population data from 2016-2018. In the study we collected secondary data from community registers from 2016 to 2018. Then we conducted the cross-sectional survey among community for members in the same study villages in 2018. Therefore Table 1. Presents findings from secondary data review while the remaining tables (table 2 and 3) presents findings from community survey. This explains the difference in the number of participants between those observed in table 1 and those from community survey.
3. **Coverage Trends Over Time and Statistical Analysis**: The statistical analysis was deployed to explore trends in the coverage for the three years period from 2016 to 2018. Since the 2019 data set was different it was nit used to compare time trends, rather to understand the drivers of drug uptake in the community. Results have been presented separately for these tow data sources in the result section of this manuscript.

**Editorial and Data Presentation Modifications?**

***Reviewer #2:***

*Title: I would consider specifying in the title that the study was conducted in Tanzania (e.g., "Mass drug administration coverage and its determinants for elimination of Onchocerciasis in Ulanga District, Tanzania).*

**Response**: Thank you for your suggestion. We agree that specifying the location in the title would improve clarity and context for readers. We have included ***"Ulanga District, Tanzania"*** to make it immediately clear where the study was conducted, which is valuable for understanding the scope and relevance of the findings.

**Abstract**

**Reviewer #2**

1. *In the abstract, is there a reason for describing age with the mode instead of the mean/median?*

**Response:** Thank you for your comment. The decision to report age using the mode in the abstract was based on the distribution of the data. We understand that the mean or median could provide a clearer understanding in many cases. We have revisited the data presentation and, reported using mean age in the abstract for better clarity and interpretation. Thanks for raising this point.

2. *The results section of the abstract do not read very well and would benefit from revision, especially ""MDA coverage for studied villages was Mawasiliano (68%, CI: 59.3 – 75.6), Uponera (83%, CI; 76.6 – 89.6), Isongo (84 .8 %, CI: 77.3 – 90.1) and Togo (79%, CI: 70.1 – 85.8).*

**Response:** Thank you for your feedback. I agree that the results section of the abstract can be improved for better readability and clarity. We have revised the sentence you mentioned to make the presentation of the coverage rates more fluid. Here’s a revised version.

" ***The study found that MDA coverage varied across villages: Mawasiliano (68%, CI: 59.3 – 75.6), Uponera (83%, CI; 76.6 – 89.6), Isongo (84 .8 %, CI: 77.3 – 90.1) and Togo (79%, CI: 70.1 – 85.8)***. ***While Uponera and Isongo achieved the WHO-recommended 80% coverage for transmission interruption, Mawasiliano and Togo fell below this threshold”***

3. *Correct punctuation in " and newcomers to the village. emphasize the need to intensify"*

**Response:** Thank you for pointing that out. To correct the punctuation and improve clarity, we have revised the entire section of the abstract to ensures proper punctuation and improves the flow of the sentence. Thanks again for your input!

4. *In the abstract, I could be beneficial information to mention that Ulanga is hyperendemic (or very endemic) for onchocerciasis prior to control, which means that indeed MDA needs to be particularly of high coverage*

**Response:** We appreciate your thoughtful feedback. You make an excellent point about providing context for the endemicity of onchocerciasis in Ulanga. Including this information would indeed highlight the importance of achieving high MDA coverage in the region for effective control. We have revised the abstract to mention that ***“Ulanga was hyperendemic for onchocerciasis before the intervention, underscoring the need for particularly high coverage in MDA efforts”.***

**Introduction**

1. *Conduct a revision for punctuation (e.g., remove commas and doubl3 spaces in "Onchocerciasis (river blindness) is a vector-borne parasitic disease caused by , Onchocerca volvulus, and...; remove unnecessary capitalisation in Onchocerciasis Control"; add "a" to "rea" in "filaria worms in the rea, suggesting").*

**Response:** Thank you for your feedback. We have revised the punctuation as suggested, to improve clarity and accuracy.

2. *In "attain and maintain the recommended MDA treatment coverage and demonstrate the interruption of transmission among vector species (6)." mention the recommended MDA treatment coverage (80% by WHO) and specify it is the coverage of the overall population.*

**Response:** Thank you for the suggestion. We have revised the sentence to include the recommended MDA treatment coverage of 80% as specified by the WHO and clarify that it refers to the coverage of the overall population.

3. *Please clarify the last part of this important sentence: ". A baseline assessment onchocerciasis conducted in 1997 indicated nodule prevalence of 45% and 95% Ulanga districts (14). "*

**Response**: Thank you for your comment. We have clarified the last part of the sentence to ensure it is more precise. The revised sentence read: ***" A baseline assessment onchocerciasis conducted in 1997 indicated nodule prevalence ranging 45% to 95% Ulanga districts*** (14)."

4. *Mention the reference used to write this sentence please "Onchocerciasis nodule prevalence was 2.3%, while the positivity rate for OV16 rapid test was 76.5% and the risk of transmission among children aged 6-9 years was 20.7%." Also, clarify what is meant by "risk", which might be misleading, if it is seroprevalence among children 6-9, then it should be stated so. Also, please specify the year of the study. Then, if the authors wish, they can state that this high seroprevalence in young children, taking into account that onchocerciasis is a chronic infection that can last over 10 years, suggests that there is still high O. volvulus transmission at that time.*

**Response:** Thank you for the helpful comments. We have revised the sentence to include the reference used for the data and clarified the meaning of "risk and specified that in the revised text. We have also included the year of the study as requested. Furthermore, we have incorporate your suggestion regarding the interpretation of the high seroprevalence, linking it to ongoing transmission of *O. volvulus*. The revised sentence will read:

"Onchocerciasis nodule prevalence was 2.3%, while the positivity rate for the OV16 rapid test was 76.5%, and the seroprevalence among children aged 6-9 years was 20.7% (reference). The study was conducted in [year]. This high seroprevalence in young children, considering that onchocerciasis is a chronic infection that can persist for over 10 years, suggests that there was still significant *O. volvulus* transmission at the time of the study."

5. *I would suggest specifying these numbers: "On the other side, literature shows the black fly carries infective parasites and the infection rate is clearly above the threshold for transmission interruption within Ulanga district (23). "*

**Response:** Thank you for the suggestion. We have specified the numbers in the sentence to make the information clearer. The revised sentence now read:

"On the other hand, literature shows that the black fly in Ulanga District carries infective parasites, with an infection rate of 0.57%, which is nearly twelve times higher than the transmission interruption threshold of 0.05% as set by the WHO (23)."

**Summary and General Comments**

**Reviewer #1:**

*I checked the responses of the authors to my comments. For me the revised paper can be published.*

**Response:** Thank you for your review and feedback. We appreciate your time and effort in evaluating our work. We are glad that the revisions have addressed your concerns, and we appreciate your support for the publication of the paper.

**Reviewer #2:**

*I congratulate the authors in addressing the reviewers' comments and adding valuable background information to their study, especially regarding onchocerciasis epidemiology in Mahenge. I believe the manuscript is in better shape to be accepted, pending minor revisions of scientific content and punctuation (e.g., double spaces, unnecessary capitalisations, missing words).*

**Response:** Thank you for your valuable feedback and kind remarks. We appreciate your acknowledgment of the revisions, particularly the additional background information on onchocerciasis epidemiology in Mahenge. We have thoroughly addressed the remaining minor revisions concerning scientific content and punctuation to ensure the manuscript is of the highest quality.

**Reviewer #3:**

*To improve validity you mentioned we used standardized tool for both survey and qualitative, how this was ensured? Which types of validity you ensured for survey also better to tell us how reliability was ensured, similarly for qualitative guide?*

**Response:** Thank you for your thoughtful comment. To ensure the validity of our study, we used standardized, pretested tools prior to data collection. For the survey, content validity was established by designing the questionnaire based on recognized frameworks and expert input, while construct validity was ensured by aligning the survey teams with the study objectives. Reliability was strengthened through internal consistency checks and pilot testing.

For the qualitative guide, validity was maintained by developing questions informed by literature and expert consultation to accurately capture key themes. Reliability was enhanced through interviewer training and data source triangulation. We have clarified these aspects in the manuscript to improve transparency.
